# Effect of mismatch repair on the mutation rate of bacteriophage ϕX174

**DOI:** 10.1093/ve/vev010

**Published:** 2015-09-10

**Authors:** Marianoel Pereira-Gómez, Rafael Sanjuán

**Affiliations:** Instituto Cavanilles de Biodiversidad y Biología Evolutiva and Departament de Genètica, Universitat de València, Paterna 46980, Spain

**Keywords:** mutation rate, bacteriophage ϕX174, methyl-directed mismatch repair, stress-induced mutagenesis, evolution

## Abstract

Viral mutation rates vary widely in nature, yet the mechanistic and evolutionary determinants of this variability remain unclear. Small DNA viruses mutate orders of magnitude faster than their hosts despite using host-encoded polymerases for replication, which suggests these viruses may avoid post-replicative repair. Supporting this, the genome of bacteriophage ϕX174 is completely devoid of GATC sequence motifs, which are required for methyl-directed mismatch repair in *Escherichia coli*. Here, we show that restoration of the randomly expected number of GATC sites leads to an eightfold reduction in the rate of spontaneous mutation of the phage, without severely impairing its replicative capacity over the short term. However, the efficacy of mismatch repair in the presence of GATC sites is limited by inefficient methylation of the viral DNA. Therefore, both GATC avoidance and DNA under-methylation elevate the mutation rate of the phage relative to that of the host. We also found that the effects of GATC sites on the phage mutation rate vary extensively depending on their specific location within the phage genome. Finally, the mutation rate reduction afforded by GATC sites is fully reverted under stress conditions, which up-regulate repair pathways and expression of error-prone host polymerases such as heat and treatment with the base analog 5-fluorouracil, suggesting that access to repair renders the phage sensitive to stress-induced mutagenesis.

## 1 Introduction

Mutation is the ultimate source of genetic variation and, therefore, a central evolutionary process. Although mutations are required for adaptation, the short-term deleteriousness of most spontaneous mutations should generally favor low mutation rates (Sniegowski et al. 2000). In theory, the balance between these short-term costs and the long-term benefits for adaptation should produce an evolutionarily optimal, intermediate mutation rate which is dependent on selection strength (Orr 2000; Johnson and Barton 2002). Other factors can also determine mutation rate evolution, including the costs of maintaining mechanisms of replication fidelity, population size, and structure, or the topology of the fitness landscape among others (André and Godelle 2006; Clune et al. 2008; Jiang et al. 2010; Lynch 2011; Sung et al. 2012). Despite this variety of factors, it has been noted that genomic mutation rates stay remarkably constant among DNA viruses, bacteria, and unicellular eukaryotes (Drake 1991; Drake et al. 1998). As a consequence, per-nucleotide rates vary by 10,000-fold and inversely with genome size, from 10^–6^ to 10^–10^ mutations per nucleotide per round of copying (m/n/r) (Lynch 2010). How evolutionary forces have shaped this inverse relationship in such widely different microbial systems and which molecular mechanisms allow for this mutation rate variation remain poorly understood questions.

For DNA viruses, mutation rates range from 10^–8^ m/n/r in double-stranded (ds) DNA viruses such as herpes virus to 10^–6^ m/n/r in single-stranded (ss) DNA viruses such as bacteriophage ϕX174, whereas these rates range from 10^–6^ to 10^–4^ m/n/r in RNA viruses (Sanjuán et al. 2010). A primary determinant of viral mutation rates is replication fidelity, and polymerase variants with altered base-selection specificities have been described in several RNA viruses including picornaviruses, alphaviruses, and retroviruses. However, fidelity variants that are not lethal typically alter mutation rates only slightly (Pfeiffer and Kirkegaard 2003; Arias et al. 2008; Menéndez-Arias 2009; Coffey et al. 2011; Graci et al. 2012). The presence of 3´exonuclease proofreading has a stronger effect on viral replication fidelity. All RNA-dependent polymerases except those of coronaviruses lack 3’ exonuclease activity, as opposed to virus-encoded DNA polymerases, therefore providing a clear basis for the higher mutation rates of RNA viruses compared with DNA viruses (Roberts, Bebenek, and Kunkel 1988; Steinhauer, Domingo, and Holland 1992; Menéndez-Arias 2009; Denison et al. 2011; Smith et al. 2013). In addition to polymerase fidelity, in DNA viruses, mutation rates should be determined by their ability to access post-replicative repair. In ssDNA bacteriophages such as ϕX174 or m13, replication is carried out by the *Escherichia coli* DNA III holoenzyme, which exhibits similar fidelity in phage and host templates (Fersht 1979; Fersht and Knill-Jones 1981). However, these phages show a mutation rate approximately three orders of magnitude higher than the host (Wickner and Hurwitz 1974; Raney, Delongchamp, and Valentine 2004; Cuevas, Duffy, and Sanjuán 2009). The high variability and fast evolution of small eukaryotic DNA viruses such as parvoviruses and polyomaviruses similarly suggests elevated rates of spontaneous mutation (Duffy, Shackelton, and Holmes 2008).

The efficiency of post-replicative repair can be higher than 99 per cent (Fijalkowska, Schaaper, and Jonczyk 2012). In *E. coli*, strand-specific bidirectional methyl-directed mismatch repair (MMR) is performed by the Dam/MutHLS system (Jiricny 2013). Point mutations or small insertion/deletion loops are recognized by MutS, which interacts with MutL, leading to activation of the MutH endonuclease. The latter recognizes the parental strand by the presence of a methyl group in the adenosine of a GATC sequence motif located on either side of the mismatch, which has been previously added by Dam methylase. MutH then cleaves the non-methylated daughter strand, which is degraded and re-synthesized (Modrich and Lahue 1996; Marti, Kunz, and Fleck 2002; Schofield and Hsieh 2003; Li 2008; Fukui 2010). Strikingly, though, the 5.4 kb genome of bacteriophage ϕX174 contains no GATC sites, whereas the randomly expected number of such sequences given the ϕX174 genome size and base composition is approximately 20. By impeding Dam methylation, the lack of GATC sites therefore avoids a major repair pathway. However, the impact of GATC motifs on the phage mutation rate is still poorly understood. In a previous study (Cuevas, Pereira-Gomez, and Sanjuán 2011), we introduced four GATC sequence motifs in the ϕX174 genome and found no effects on mutation rate. However, by increasing the number of GATC motifs to seven, we obtained a thirtyfold reduction in mutation rate. Furthermore, this effect was reverted in MMR-deficient *mutD* cells, indicating that the effects of GATC motifs were related to MMR.

Here, to better explore how MMR avoidance determines the mutation rate of ϕX174, we constructed a ϕX174 variant encoding twenty randomly located GATCs with minimal effects on protein sequence. The engineered phage showed an eightfold reduction in spontaneous mutation rate compared with the wild type (WT), yet no obvious growth defects under standard conditions. However, the efficacy of GATC-driven MMR was curtailed by poor methylation of the phage DNA, preventing recognition of the parental strand. Furthermore, after constructing several mutants in which the number and location of GATC sites were varied, we found that their effects on mutation rate were non-additive and highly variable, with some combinations achieving an up to fiftyfold reduction in mutation rate while others having no effects. The highest efficiencies were shown by some intergenic GATCs, suggesting that steric constrains such as availability of the DNA to MutH may be important for MMR. Finally, we found that the mutation rate reduction afforded by the twenty GATC motifs was fully reverted at 42°C and in the presence of the base analog 5-fluorouracil (5-FU), two stress factors that promote overexpression of repair-associated error prone polymerases (Layton and Foster 2005; Malkova and Haber 2012), thus suggesting that addition of GATC motifs renders the phage sensitive to stress-induced mutagenesis.

## 2 Materials and methods

### 2.1 Bacteriophage and cells

The *E. coli* C strain IJ1862 was obtained from Prof. James J. Bull. The *gro87* mutant was provided by Prof. Bentley A. Fane (University of Arizona). Bacteriophage ϕX174 originally obtained from Prof. James J. Bull (Texas University) was adapted to our laboratory conditions by long-term passaging in IJ1862 cells (Domingo-Calap, Cuevas, and Sanjuán 2009). GATC sites were engineered in the genetic background of this adapted virus, here denoted the WT, which contains no GATCs (GenBank accession GQ153915).

### 2.2 Site-directed mutagenesis

The ϕX174 dsDNA replicative form was purified from infected cultures before lysis using a standard miniprep kit (Macherey-Nagel), and 500 pg of this DNA were used as template for polymerase chain reaction-based mutagenesis using Phusion high-fidelity DNA polymerase (Thermo Scientific) and contiguous, divergent, 5’-phosphorylated primers, of which the reverse primer carried the desired nucleotide substitution. Polymerase chain reaction products were circularized with the Rapid DNA ligation kit (Thermo Scientific) and used for transfecting competent IJ1862cells by the classical heat-shock method. A single plaque was picked, resuspended Lysogeny Broth medium, and stored at –70°C. The presence of each substitution was confirmed by Sanger sequencing. This process was iterated until twenty GATC sites were introduced. Full-length sequencing of the 20GATC virus was performed to verify that all mutations were present and that no other changes were introduced.

### 2.3 Luria–Delbrück fluctuation tests

Each test consisted of twenty-four independent 0.5 ml IJ1862 cultures inoculated with the indicated initial number (*N*_0_) of plaque forming units (pfu) and incubated in a Thermomix shaker (Eppendorf) at 650 rpm until *N*_1_ pfu were produced. This growth phase was done under standard conditions (37°C), at high temperature (42°C), or in the presence of 10 ng/µl 5-FU (37°C) by pre-incubating cells with 5-FU 10 min before infection. All titrations were done under the same, standard conditions (IJ1862 cells with agar overlay, 37°C, no 5-FU). *N*_1_ was determined by titrating six of twenty-four random cultures. To score mutants, 0.4 ml (80% of the total volume) was titrated on the restrictive *E. coli gro87* strain, a *rep* mutant where only ϕX174 mutants with certain mutations in the N-terminal end of the viral protein A can form plaques (Ekechukwu, Oberste, and Fane 1995), the total number of different substitutions leading to the resistance phenotype being *T* = 7 under our assay conditions (Cuevas, Duffy, and Sanjuán 2009). We estimated the rate *m* at which *gro87*-resistant mutants appeared using the null-class method, which is based on counting the proportion of cultures showing zero versus at least one mutant. The number of mutations per culture should follow a Poisson distribution with parameter *λ* = *m*(*N*_1_ – *N*_0_), such that the expected probability of no mutants in a culture is *P*_0_ = exp[–*m*(*N*_1_ – *N*_0_)]. Mutation rates per nucleotide per round of copying (m/n/r) were then calculated as *µ* = 3 m/T, where the factor 3 stands for the fact that each base can mutate to three different bases. Three independent tests were performed for each mutant, except for the WT, for which fifteen tests were performed. Mutation rate estimates for the WT in the presence of 5-FU 10 ng/µl were taken from a previous work (Pereira-Gómez and Sanjuán 2014). For fluctuation tests performed under stress conditions, we applied a correction for bias in *N*_1_ estimation which may result from increased viral degradation relative to standard conditions. Following previous work (Bradwell et al. 2013), the probability of observing no mutants was recalculated accordingly as *P*_0_ = exp[–*m*(*N*_1_/*z*´– *N*_0_)], where *z*´ quantifies this bias. As an indicator of *z*´, we determined the relative plating efficiency of the WT virus under the two stress conditions. Plating efficiency was slightly increased at 42°C (*z*´ = 1.13 ± 0.13) and reduced in the presence of 10 ng/µl 5-FU (*z*´ = 0.75 ± 0.12). We therefore used the corresponding *z*´ values for calculating mutation rates at 42°C and in the presence of 5-FU. Since the relative plating efficiencies of the 20GATC virus did not differ significantly from those of the WT (*t*-test: *P* > 0.5), we used the same *z*´ values. Q-Q plots showed that mutation rate estimates were not normally distributed, whereas normality was satisfied using log-transformed rates. All statistical tests were thus performed using log-transformed rates.

### 2.4 Growth rate estimation

The viral exponential growth rate was estimated as *r* = ln(*N*_1_/*N*_0_)/*t*, where *N*_1_ and *N*_0_ were obtained from the fluctuation test assays, and *t* is the incubation time in hours. Q-Q plots indicated that growth rates were normally distributed.

### 2.5 Methylation analysis

The ϕX174 dsDNA replicative form was quantified using the Quant-iT PicoGreen dsDNA broad range assay kit (Life technologies), and all extracts were brought to the same concentration (30 ng/µl). DNA from each virus was split into three aliquots, which were treated with XhoI to linearize the genome, with XhoI and DpnI to digest methylated GATCs, or with XhoI and MboI (i.e., DpnII) to digest non-methylated GATCs. Double digestions were performed according to the manufacturer instructions (Thermoscientific). A standard plasmid (pIRES, Clontech) was used as a digestion control (not shown). A monochrome picture of the gel was transformed to an eight-bit image, and the pixel area and intensity of each band were quantified using ImageJ.

## 3 Results

### 3.1 Introduction of GATC sites reduces the ϕX174 mutation rate

**Figure 1.**
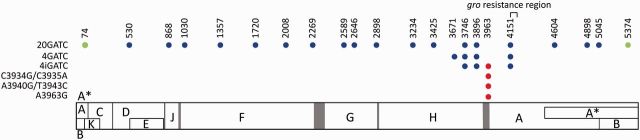
ϕX174 genetic map and location of the GATC sequence motifs introduced in this study. Open reading frames are represented by rectangles (B, K, and E are in different reading frames), and gray bars indicate intergenic regions. Each GATC is represented by a dot, and its position is indicated on top. GATC motifs that were synonymous in all reading frames are indicated in blue, whereas those producing amino acid replacements in at least one frame are shown in green (A74C produces a K494Q replacement in gene A and is synonymous in gene K; T5374G produces a V465G replacement in gene A and is synonymous in gene B). Mutations falling at intergenic regions are shown in red. The phage has circular DNA but is represented linearly for convenience, where by convention the first position corresponds to the last nucleotide of the unique PstI site.

Given the size and base composition of the ϕX174 DNA (5,386 bases, 31.3% T, 24.0% A, 23.2% G, and 21.5% C), the expected number of GATC sequence motifs in its genome is 0.313 ×  0.240 × 0.232 × 0.215 × 5,386 = 20.18. The Poisson probability of observing no GATC motifs is extremely low (*P* = 1.72 × 10^–9^), thus indicating a strong avoidance of these motifs. To restore GATC usage in ϕX174, we created a mutant phage carrying twenty GATCs by sequential addition of these sites into the WT virus using site-directed mutagenesis (Fig. 1).

**Figure 2.**
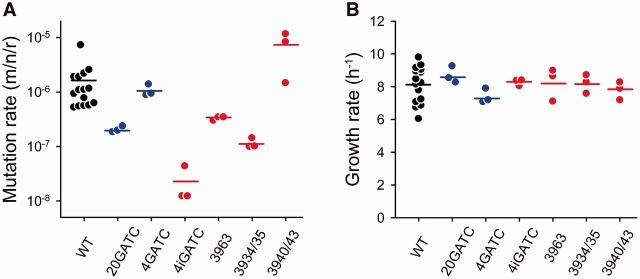
Effect of different GATC sequence motifs on the mutation rate and growth rate of ϕX174. Mutation rates estimated by the Luria–Delbrück fluctuation test (A) and growth rates obtained in these same assays (B) for the WT virus (*n* = 15) and GATC mutants (*n* = 3) are shown. gATC mutants containing only synonymous substitutions are shown in blue, whereas those containing at least one intergenic substitution are shown in red. Each dot represents an individual estimate and horizontal bars indicate the mean. See Table 1 for details.

The twenty GATC motifs were evenly distributed throughout the phage genome, the greatest distance between any two consecutive of them being 456 bases and, wherein possible, substitutions were made synonymous to minimize their effects on protein function. Given that the Dam/MutHLS system can perform MMR at a distance of up to 1 kb from a GATC (Modrich and Lahue 1996), the number and distribution of the introduced GATCs should allow for efficient MMR in the entire phage genome. To test the effect of GATCs on the viral mutation rate, we performed Luria–Delbrück fluctuation tests for the WT and 20GATC viruses. To score mutants phenotypically, we used the non-permissive *E. coli* C mutant *gro87*, which carries a mutation in the DNA helicase gene *rep* that blocks stage III ssDNA synthesis, preventing maturation of the WT phage (Ekechukwu, Oberste, and Fane 1995). The phage can overcome this restriction by changing certain amino acid residues in the N-terminal region of protein A, an endonuclease that nicks the negative strand of the supercoiled phage DNA, and these protein changes can be conferred by at least seven different nucleotide substitutions (Cuevas, Duffy, and Sanjuán 2009). By growing the virus in permissive cells and performing plaque assays in *gro87* cells to score mutations, we obtained an estimated mutation rate for the WT of (1.58 ± 0.44) × 10^–6^ m/n/r, a value consistent with previous studies (Raney, Delongchamp, and Valentine 2004; Cuevas, Duffy, and Sanjuán 2009). In contrast, the rate of the 20GATC virus was (2.06 ± 0.15) × 10^–7^ m/n/r, revealing a 7.7-fold reduction compared with the WT (*t*-test: *P* < 0.001; Table 1; Fig. 2A). The estimated growth rate was similar for the WT (*r* = 8.11 ± 0.29 h^–1^) and the 20GATC viruses (*r* = 8.68 ± 0.29 h^–1^; *t*-test: *P* = 0.406; Table 1; Fig. 2B), indicating that these substitutions had no significant impact on short-term viral fitness. These findings confirm our previous results obtained with a mutant phage carrying seven GATC sites (Cuevas, Pereira-Gomez, and Sanjuán 2011).

**Table 1.** Fluctuation test data (mean ± SEM) of ϕX174 WT and GATC mutants.

Virus	Tests^a^	*N*_0_^b^	*N*_1_^c^^ ^× 10^–6^	*P*_0_^d^	*m*^e^^ ^× 10^6^	*µ*^f^^ ^× 10^6^	*r*^g^
WT	15	211 ± 25	0.28 ± 0.05	0.52 ± 0.05	3.69 ± 1.03	1.58 ± 0.44	8.11 ± 0.29
20GATC	3	320 ± 134	3.09 ± 1.03	0.29 ± 0.13	0.48 ± 0.04	0.21 ± 0.15	8.68 ± 0.29
4GATC	3	117 ± 16	0.11 ± 0.00	0.76 ± 0.05	2.50 ± 0.37	1.07 ± 0.16	7.38 ± 0.26
4iGATC	3	128 ± 3	4.06 ± 1.10	0.74 ± 0.11	0.08 ± 0.02	0.03 ± 0.01	8.27 ± 0.11
A3963G	3	197 ± 30	1.15 ± 0.73	0.54 ± 0.23	0.79 ± 0.05	0.33 ± 0.02	8.24 ± 0.58
C3934G/C3935A	3	326 ± 76	1.17 ± 0.31	0.75 ± 0.04	0.27 ± 0.03	0.12 ± 0.01	8.19 ± 0.33
A3940G/T3943C	3	395 ± 207	0.21 ± 0.16	0.35 ± 0.20	16.6 ± 6.95	7.13 ± 2.98	7.78 ± 0.32

^a^Each test consists of twenty-four independent cultures.

^b^Initial number of pfu per culture.

^c^Final number of pfu per culture.

^d^Fraction of cultures showing no *gro87* resistant plaques.

^e^*gro*-resistance mutation rate estimated by the null-class method as $m=\;\frac{-\text{ln}P_{0}}{N_{1}-N_{0}}$.

^f^$µ=\;\frac{3m}{T}$, where *T* = 7 is the number of substitutions leading to *gro87* resistance.

^g^$r=\;\text{ln}(N_{1}/N_{0})/t$, where *t* is the incubation time in hours.

### 3.2 The effect of GATC sites is limited by under-methylation of the phage DNA

**Figure 3.**
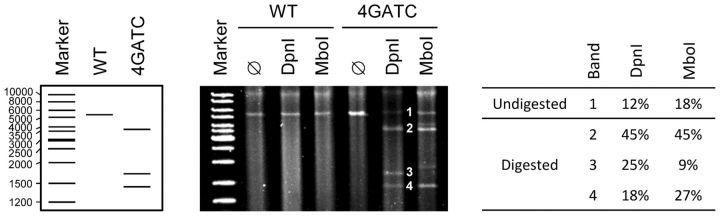
Restriction fragment analysis of the ϕX174 replicative dsDNA. Phage dsDNA was purified by standard miniprep as described in the Materials and Methods section and linearized with XhoI (ø), which recognizes a unique site at position 162, with XhoI and DpnI to cleave methylated or hemi-methylated GATCs, or with XhoI and MboI to cleave non-methylated GATCs. Expected (left) and observed (center) restriction fragments for the WT and 4GATC phage dsDNA are shown. Lower size fragments (<300 bp, Fig. 1) were expected but could not be visualized because the amount of input DNA was low. The smear in lanes containing the purified phage DNA probably results from degradation of host DNA. The contrast of the gel image was enhanced to help visualize bands. Right: percent abundance of each DpnI and MboI restriction band (1–4). Band 1 in the DpnI lane indicates the non-methylated DNA fraction (i.e., none of the four GATC motifs was methylated), whereas in the MboI lane, this same band indicates the fully methylated fraction (i.e., the four GATC motifs were methylated). Bands were quantified as detailed in the Materials and Methods section using the raw gel image with no contrast enhancement.

Since MMR relies on the presence of GATCs in the vicinity of the mismatch, addition of GATCs in this region should suffice to yield similarly low mutation rates. On the basis of this, we constructed a virus with four GATCs located between genome positions 3671 and 4151, which were within 0.5 kb of known *gro*-resistance mutations (Fig. 1). However, surprisingly, the mutation rate of this four GATC virus was significantly higher than that of the 20GATC virus (*t*-test: *P* = 0.001) and similar to the WT rate (*P* = 0.832). In light of this result and since Dam methylation of GATC adenosines is required for MMR, we sought to determine the methylation status of the 4GATC ϕX174 DNA. To do so, we purified the dsDNA replicative form, linearized it, and treated it with the DpnI restriction endonuclease, which selectively digests methylated and hemi-methylated GATCs. Although DpnI produced restriction bands of the expected size, digestion was only partial (Fig. 3). To test whether this could be explained by incomplete DNA methylation, we performed the same restriction analysis using MboI, which also recognizes GATC sites but digests them only in their non-methylated form. MboI also produced restriction bands, thus confirming that a fraction of the phage dsDNA was not methylated. Image analysis indicated that 82 per cent of DNA was digested by MboI and thus lacked at least one of the four possible methyl groups, whereas 12 per cent was undigested by DpnI, thus lacking all four methyl groups. Overall, the similar efficiency shown by DpnI and MboI suggests that roughly half of GATC motifs were methylated, although more detailed analyses would be required to reliably infer this fraction. We verified that under-methylation was not due to a Dam defect in the host cell, since a standard plasmid grown in the same *E. coli* strain was fully digested by DpnI and fully resistant to MboI (not shown). Therefore, these results suggest that, as opposed to plasmid or chromosomal DNA, GATC-mediated MMR is not fully efficient in ϕX174 because the phage DNA is under-methylated.

### 3.3 Intergenic GATC sites have highly variable effects on the phage mutation rate

Given that protein-coding regions represent approximately 95 per cent of the ϕX174 genome, by chance one in twenty 20 GATC sites should fall at intergenic regions. To test how GATC location may influence MMR and the phage mutation rate, we created another mutant (4iGATC) in which one of the four synonymous substitutions of the above 4GATC virus was replaced by substitution A3963G, which was located in the spacer region between genes H and A. The mutation rate of the 4iGATC was (2.27 ± 1.04) × 10^–8^ m/n/r, which represents a fiftyfold reduction compared with the WT (*t*-test: *P* < 0.001; Table 1). Therefore, addition of this single intergenic substitution had a dramatic effect on the viral mutation rate, compared with the 4GATC virus. To further test the effect of intergenic GATCs on MMR, we first created the single mutant A3963G, which showed a mutation rate five times lower than the WT (*t*-test: *P* < 0.001; Table 1). This rate was significantly higher than for the 4iGATC virus (*t*-test: *P* = 0.020), showing that the effect of the A3963G substitution was enhanced by the presence of other, neighboring GATC sites, consistent with the lack of full methylation shown above. Then, we constructed two additional intergenic single-GATC viruses located in the region between the H and A genes by introducing the appropriate nucleotide substitutions (Fig. 1). The mutation rate of the virus carrying the C3934G/C3935A substitutions was fourteen times lower than the WT (*t*-test: *P* < 0.001; Table 1), whereas substitutions A3940G/T3943C were unable to reduce the mutation rate below the WT level (*P* = 0.132) despite being located only five bases away from the previous substitutions. Therefore, some but not all intergenic GATCs are able to promote MMR, and minute changes in their genome location lead to marked differences in mutation rate.

### 3.4 The effect of GATC motifs on the phage mutation rate is reverted under stress

**Figure 4.**
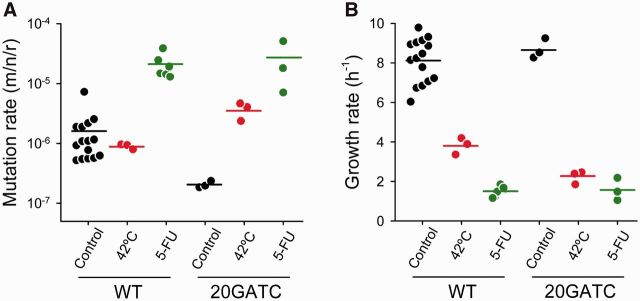
Mutation rates and growth rates in the presence of stress factors. Mutation rates estimated by the Luria–Delbrück fluctuation test (A) and growth rates obtained in these same assays (B) are shown for the WT (*n* = 15) and the 20GATC virus (*n* = 3–6) in the presence of thermal stress (red), 5-FU (green), or under control conditions (black; from Fig. 2). Each dot represents an individual estimate and horizontal bars indicate the mean. See Table 2 for details.

Induction of the chaperone-encoding *groE* operon in response to heat shocks up-regulates the expression of the error-prone DNA polymerase IV, which participates in the repair of dsDNA breaks under different types of cellular stress, leading to stress-induced mutagenesis (Layton and Foster 2005; Malkova and Haber 2012). To address the effects of heat shocks on the ϕX174 mutation rate, we performed fluctuation tests at 42°C for the WT and 20GATC viruses. The mutation rate of the WT was not significantly affected by the temperature shift (*t*-test: *P* = 0.585; Table 2; Fig. 4A). In contrast, the mutation rate of the 20GATC virus was twenty times higher at 42°C than at 37°C (*t*-test: *P* < 0.001) and increased even above the WT level (*P* = 0.003). Therefore, the effects of GATC sites on the ϕX174 mutation rate observed at 37°C were reverted at 42°C.

**Table 2.** Fluctuation test data (mean ± SEM) of the WT and 20 GATC viruses under stress.

Virus	Stress	Tests	*N*_0_	*N*_1_^a^^ ^× 10^–6^	*P*_0_	*m* × 10^6^	*µ* × 10^6^	*r*
WT^b^	None	15	211 ± 25	0.28 ± 0.05	0.52 ± 0.05	3.69 ± 1.03	1.58 ± 0.44	8.11 ± 0.29
WT	42°C	3	187 ± 22	5.18 ± 1.58	0.38 ± 0.11	2.10 ± 0.12	0.90 ± 0.06	3.81 ± 0.24
WT^c^	5-FU	6	273 ± 58	0.01 ± 0.00	0.53 ± 0.11	48.8 ± 9.45	20.9 ± 4.12	1.50 ± 0.11
20GATC^a^	None	3	320 ± 134	3.09 ± 1.03	0.29 ± 0.13	0.48 ± 0.04	0.21 ± 0.15	8.68 ± 0.29
20GATC	42°C	3	133 ± 19	0.38 ± 0.15	0.71 ± 0.13	9.79 ± 1.81	3.72 ± 0.69	2.23 ± 0.19
20GATC	5-FU	3	109 ± 36	0.03 ± 0.03	0.60 ± 0.28	59.5 ± 30.8	25.5 ± 13.2	1.57 ± 0.33

^a^Corrected for differences in plating efficiency at 42°C and 5-FU (see Materials and Methods).

^b^From Table 1.

^c^From a previous work (see Materials and Methods).

Heat drastically reduced the viral growth rate but, whereas the WT and 20GATC viruses showed similar growth rates at 37°C, the 20GATC virus grew significantly slower than the WT at 42°C (2.23 ± 0.19 h^–1^ versus 3.81 ± 0.24 h^–1^; *t*-test: *P* = 0.007; Table 2; Fig. 4B), suggesting that up-regulation of repair pathways under thermal stress slows down phage replication. To evaluate the effects of another stressor, we treated cells with 5-FU (10 ng/µl) which, in addition to causing mutations directly by base mispairing, 5-FU inhibits thymidylate synthase, leading to deoxythymidine monophosphate deprivation and, subsequently, to DNA strand breaks, induction of the SOS DNA damage response (DDR), and expression of error-prone DNA repair enzymes (Ahmad, Kirk, and Eisenstark 1998; Fonville et al. 2010). Previously, we showed that this treatment increases the mutation rate of the WT virus by approximately tenfold (Domingo-Calap, Pereira-Gomez, and Sanjuán 2012). Fluctuation tests in the presence of 10 ng/µl 5-FU showed that the drug had a more pronounced effect on the mutation rate of the 20GATC virus, which increased more than a 100-fold (Table 2; Fig. 4A). As a result, the difference in mutation rate between the 20GATC and WT viruses was fully abolished in the presence of 5-FU (*t*-test: *P* = 0.951).

## 4 Discussion

We have shown that introduction of GATC sites in the ϕX174 genome can reduce the spontaneous mutation rate of the phage by up to fiftyfold, indicating that phage DNA can undergo MMR if the required sequence motifs are present. The effect of GATC addition is greater than those reported previously in RNA viruses, in which high-fidelity polymerase variants selected after serial transfers in the presence of nucleoside analogs typically reduce the viral mutation rate by threefold or less (Pfeiffer and Kirkegaard 2003; Coffey et al. 2011). A similarly modest effect was observed after serial passaging of ϕX174 in the presence of 5-FU (Domingo-Calap, Pereira-Gomez, and Sanjuán 2012). In that case, the anti-mutator phenotype was achieved by a delayed lysis, which increased the viral burst size per cell and thus allowed the phage to expand its population size in fewer rounds of copying (Pereira-Gómez and Sanjuán 2014). In the dsDNA bacteriophage T4, a series of polymerase variants capable of strongly suppressing the action of chemical mutagens were isolated in early studies (Drake et al. 1969; Drake and Greening 1970). However, high fidelity variants of T4 polymerase tend to show diminished polymerization rates, therefore negatively impacting viral fitness (Mansky and Cunningham 2000). In *E. coli*, changes in the α subunit of DNA polymerase III can increase replication fidelity between two- and thirtyfold (Fijalkowska, Dunn, and Schaaper 1993). A stronger anti-mutator phenotype was found in the adenine-dependent *E. coli mud* strain, but latter analyses suggested that this was probably due to poor detection of mutants (Schaaper 1998). In another study, *E. coli* clones were isolated with an up to fiftyfold anti-mutator phenotype, but the underlying mechanisms remained undetermined (Quinones and Piechocki 1985). Therefore, the magnitude of the mutation rate reduction afforded by the introduction of GATC motifs is similar or higher than those reported previously in other viruses and in bacteria and has a well-defined molecular basis.

Our results suggest that the ϕX174 mutation rate can be modified without significantly impacting viral fitness in the short-term, therefore allowing for evolutionary optimization of the viral mutation rate for long-term adaptability. However, our results also revealed constraints limiting mutation rate evolution, since the effects of GATC addition were lower than expected if MMR were fully efficient (Fijalkowska, Schaaper, and Jonczyk 2012). Illustrating this, the lowest mutation rate achieved in this study (3 × 10^–8^ m/n/r) was still two orders of magnitude higher than that of *E. coli* (Drake 1991; Drake et al. 1998; Lee et al. 2012). Our results suggest that inefficient MMR in ϕX174 is at least in part due to the fact that phage DNA is under-methylated. Full methylation may be impeded by the fast replication of the phage and the transient nature of the dsDNA replicative form. Cellular Dam methylase levels must be tightly regulated, because hypo- and hypermethylation can compromise the ability of the MMR system to distinguish between the parental and daughter DNA strands. Showing this, both Dam deficiency (Bale, d'Alarcao, and Marinus 1979; Marinus 2010) and overexpression (Pukkila et al. 1983; McClelland 1984) have been found to produce mutator phenotypes in *E. coli*. It is possible that Dam methylation levels which are optimal for the host are too low for the phage, due to its fastest replication. This could be tested in future work by infecting Dam-overexpressing *E. coli* C mutants with the 20GATC phage and determining phage DNA methylation levels and mutation rates. Assuming that the MMR system can use GATC sites at a distance of up to 1 kb from the mismatch, for a DNA showing the randomly expected density of GATCs, there should be approximately four such sites available for each mismatch. For a 50 per cent methylation efficiency, the fraction of non-reparable mismatches would thus be on the order of 0.5^4 ^= 0.06, implying that the maximum mutation rate reduction achievable by MMR for this methylation efficiency would be 1/0.06 = seventeenfold. Incomplete GATC methylation can hence account for the relatively limited efficacy of MMR in ϕX174. We note that the fiftyfold mutation rate reduction observed for the 4iGATC virus could be achieved with 63 per cent methylation, a value experimentally undistinguishable from the 50 per cent assumed above given that we could not finely quantify the fraction of methylated DNA.

However, for some GATCs, the efficacy of MMR was clearly below the upper-limit imposed by under-methylation, since the 4GATC and A3940G/T3943C viruses showed no change in mutation rate at all. Our results indicate that intergenic GATCs tended to have stronger effects than those located in protein-coding regions, suggesting other factors curtailing MMR efficiency such as steric availability to MutH. As we have shown, though, even two extremely close GATCs can have very different effects on mutation rate, and we lack a model for explaining these differences. The region in which the single intergenic GATCs were placed contains the A promoter and the H terminator. The C3934G/C3935A substitution, which had the strongest effect on mutation rate, was located farthest away from the A promoter. We can speculate that, if steric availability of the GATC motif to MutH was limited by the transcription machinery, a more distal positioning from actively transcribed regions may allow for more efficient MMR. However, the A3963G and A3940G/T3943C substitutions were located approximately at the same distance of the A promoter. A3940G/T3943C was upstream of the promoter, whereas A3963G was downstream of the promoter and in a palindromic region with a relative high G+C content. As suggested previously, mismatch recognition may depend also on sequence context, increasing its efficiency in regions with higher GC content (Jones, Wagner, and Radman 1987).

Therefore, our results suggest that evolutionary optimization of the mutation rate may not be the sole factor driving GATC avoidance in ϕX174 or other enterobacteriophages, since we found that some GATCs had no effect on the viral mutation rate yet are also absent from the WT ϕX174 genome. Mutation rate elevation can confer faster adaptation to new and stressful environments, and this has been shown to promote the spread of mutator strains in bacteria (LeClerc et al. 1996; Sniegowski, Gerrish, and Lenski 1997; Jolivet-Gougeon et al. 2011). Furthermore, bacteria have evolved the ability to up-regulate their mutation rates in response to stress by expressing of error-prone polymerases (Rosenberg 2001; Galhardo, Hastings, and Rosenberg 2007). However, we have shown that GATC avoidance does not appear to increase the ϕX174 mutation rate under stress conditions, thus undermining the potential evolutionary advantage of such avoidance. It has been shown that phage yields tend to decrease in *dam^–^ mutH^+^* suggesting that, in the absence of methylation, MutH cleaves some GATC sites non-specifically and may also interfere with other stages of the infection cycle such as replication or encapsidation (Deschavanne and Radman 1991). This would directly counter-select GATC sequence motifs in the phage. Although in our experimental setting, we did not detect a significant deleterious fitness effect associated with GATCs in the absence of stress, such effects may potentially take place in other environments not assayed here. Therefore, the evolutionary forces shaping GATC avoidance remain unclear, and may result from the joint action of several factors.

Interestingly, DNA repair pathways may also be relevant to virus-host interactions in eukaryotes. Vertebrate DNA viruses have been shown to interact with the evolutionarily conserved DDR, which is aimed at detecting lesions in DNA, initiating cell cycle arrest, and promoting repair. For instance, in hepadnaviruses, the synthesis of replication-competent covalently closed circular DNA requires the participation of Ku80, a component of non-homologous end joining DNA repair pathway (Guo et al. 2012). Indeed, DNA damage induction seems to be a common feature of many DNA viruses including adenoviruses, herpesviruses, polyomaviruses, and papillomaviruses (Luftig 2014). Most viruses degrade DDR components, but DDR activation and recruitment of some of its components into viral replication centers is also common. However, the outcomes of virus-host interactions at the DDR level are still poorly understood, and it remains at present unknown whether DDR activation is part of an antiviral cellular response or is exploited by the virus. In any case, repair pathways are activated following virus-induced DNA damage, leading to the recruitment of error-prone host polymerases (Malkova and Haber 2012). This suggests that changes in MMR efficiency following infection may determine the mutation rates of some DNA viruses.
